# Bioassay-Guided Alkaloids Isolation from *Camellia sinensis* and *Colchicum luteum*: *In Silico* and *In Vitro* Evaluations for Protease Inhibition

**DOI:** 10.3390/molecules28062459

**Published:** 2023-03-08

**Authors:** Mohammad Aatif, Muhammad Asam Raza, Mohamed El Oirdi, Mohd Farhan, Muhammad Waseem Mumtaz, Muhammad Hamayun, Adnan Ashraf, Ghazala Muteeb

**Affiliations:** 1 Department of Public Health, College of Applied Medical Sciences, King Faisal University, Al Ahsa 31982, Saudi Arabia; 2 Department of Chemistry, Hafiz Hayat Campus, University of Gujrat, Gujrat 50700, Pakistan; 3 Department of Basic Sciences, Preparatory Year Deanship, King Faisal University, Al Ahsa 31982, Saudi Arabia; 4 Department of Chemistry, University of Lahore, Lahore 53700, Pakistan; 5 Department of Nursing, College of Applied Medical Sciences, King Faisal University, Al Ahsa 31982, Saudi Arabia

**Keywords:** bioactive compounds, medicinal chemistry, purification, docking studies, pharmaceutical assessment

## Abstract

Bioassay-guided isolation from *Camellia sinensis* (Theaceae) and *Colchicum luteum* (Liliaceae) utilizing an in vitro model of protease assay revealed colchicine (**1**) and caffeine (2) from chloroform fractions, respectively. Their structures were validated using spectral techniques. The purified compounds were further optimized with Gaussian software utilizing the B3LYP functional and 6-31G(d,p) basis set. The result files were utilized to determine several global reactivity characteristics to explain the diverse behavior of the compounds. Colchicine (**1**) showed a higher inhibition of protease activity (63.7 ± 0.5 %age with IC_50_ = 0.83 ± 0.07 mM), compared with caffeine (**2**) (39.2 ± 1.3 %age). In order to determine the type of inhibition, compound **1** was further studied, and, based on Lineweaver–Burk/Dixon plots and their secondary replots, it was depicted that compound **1** was a non-competitive inhibitor of this enzyme, with a Ki value of 0.690 ± 0.09 mM. To elucidate the theoretical features of protease inhibition, molecular docking studies were performed against serine protease (PDB #1S0Q), which demonstrated that compound **1** had a strong interaction with the different amino acid residues located on the active site of this understudied enzyme, with a high docking score of 16.2 kcal/mol.

## 1. Introduction

Nature is a one-of-a-kind and less expensive source of therapeutic plant-derived products for a wide range of ailments [1]. Medicinal plants are a large reservoir for the pharmaceutical sector, which is involved in the development of various pharmaceuticals for the benefit of the population [1]. They are mostly composed of bioactive chemicals that have been used in the medical profession throughout history [2]. Several hundred species are employed for the cure of different disorders in the indigenous systems of medicine in the form of whole plants or their extracts [3,4,5]. The use of plants, their various parts, and their extracts, as well as purified chemicals from natural sources, is increasing day by day due to the heavy cost and side effects of synthesized compounds. These discoveries have laid the groundwork for modern therapeutic sciences and made it possible for people to create a system of medicine based on evidence [6]. Secondary metabolites with divergent and complicated structures are found in plants. Numerous plants include pharmacological moieties such as alkaloids, saponins, tannins, cardiac glycosides, and polyphenols, which account for more than 30% of commercial pharmaceuticals worldwide [7]. 

Plants can defend themselves against pests by manufacturing particular macromolecules in the form of enzyme inhibitors such as protease [8], amylase [9], lectins, and pectinase inhibitors [10]. In our body, each biochemical transformation is catalyzed with the help of a specific enzyme. So, we can say that the function of our body is fully controlled by different enzymes. When there are irregularities in their function, various problems may arise that lead to different diseases. Proteases are a class of enzymes whose catalytic purpose is to hydrolyze peptide bonds. They have an important role in a range of biological processes, both physiological and infectious [11,12]. Proteases are classified into five major classes based on their different catalytic actions: serine, threonine, cysteine, aspartate, and metallo proteases. Among all these proteases, serine, metallo, and cysteine are the most abundant in human beings. These are studied most extensively in various researched groups of the proteases [13]. Trypsin cleaves polypeptide chains on the C-terminus of arginine- or lysine-containing positively charged side chains [14].

Over the past decades, the advancement of technology and theoretical understanding, as well as in silico approaches, have been essential for researchers, especially in the medical field [15]. These factors can be used to predict numerous factors relating to the physical and chemical behavior of molecules. In the medical industry, information about medicine, such as the property of an isolated or manufactured molecule, can be appraised using various software. The most adaptable and dependable strategy to generate or design innovative pharmaceuticals in the form of therapeutic agents or inhibitors with good biological response in terms of ligand–protein (receptor) binding interactions is molecular modeling (enzyme). These interactions help to improve understanding of the molecule’s activity at the active site of the enzyme, which is necessary for disease therapy [1].

Following a thorough review of the literature [16,17], we chose two medicinal plants (*Camellia sinensis* and *Colchicum luteum*) for their phytochemistry and ethnobotanical applications. Green tea leaves (*Camellia sinensis*) are a member of the Theaceae family. Theaceae are widely used as antioxidants in beverages and foods and are considered the world’s second most popular drink after water. They are also used as anti-inflammatory, anti-nervous system, blood sugar reducer, and anti-cancer agents [16,17,18]. Green tea is a promising treatment option for patients with coronary heart disease, hyperlipidemia, and arteriosclerosis [19]. It contains a variety of polyphenols and tannins, with the main ingredients being epigallocatechin gallate (EGCG), epicatechin gallate (ECG), epigallocatechin (EGC), epicatechin (EC), and catechin. Green tea catechins are made up of 50–60% EGCG [20,21]. *Colchicium luteum* (family; Liliaceae), also known locally as ‘Suranjan-e-Talkh’ [22], has 31 different alkaloids, the structures of which have been reported, and colchicine is the most abundant bioactive compound among these alkaloids [23]. It is used to treat jaundice, rheumatism, colds, coughs, wounds, eruptions, joint discomfort, leucorrhea, and other ailments. *C. luteum* has antifungal properties as well as the ability to inhibit acetylcholinesterase, butyrylcholinesterase, lipoxygenase, and urease [24,25]. The main goal of this study was to isolate the major bioactive components of these selected medicinal plants and test them as protease inhibitors *in vitro* and *in silico*.

## 2. Results and Discussion

### 2.1. Extraction and Purification of Bioactive Compounds

The plant material was collected, dried, powdered, and extracted in ethanol, yielding a crude extract that was polarity partitioned with several solvents. The crude extract and various fractions generated after partitioning were tested for enzyme inhibition using the published method [25], and the results are displayed in Table 1. It was found that ethanolic extract exhibited an inhibition of 49.7 ± 1.1 %age, n-hexane showed a weak response, and the chloroform fraction at pH 9.0 depicted maximum enzyme inhibition activity (54.1 ± 0.5 %age).

**Scheme 1:**
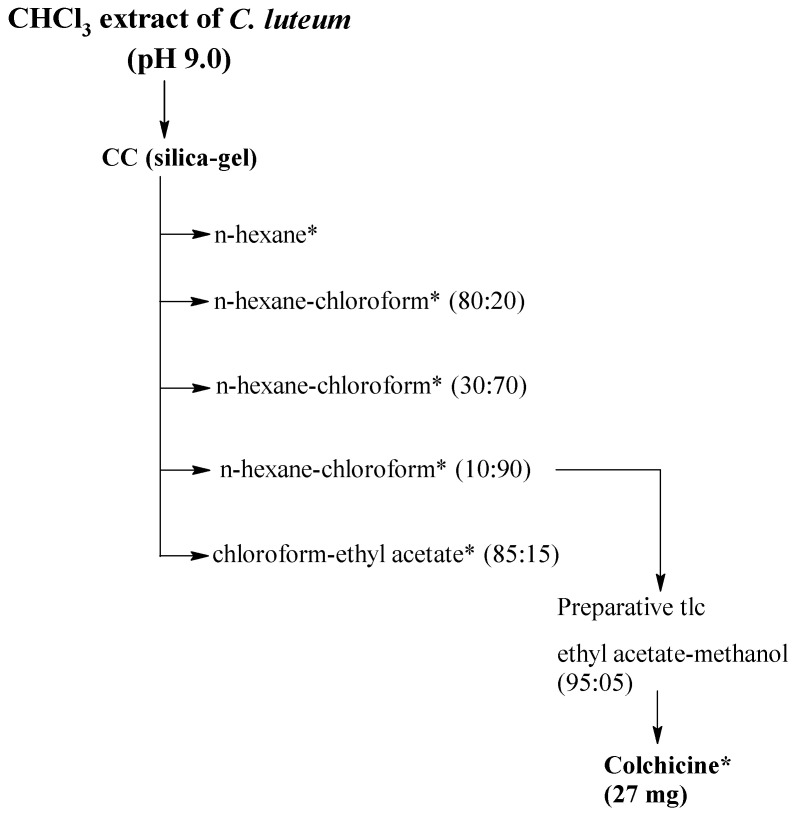
Isolation and purification of colchicine (**1**). * = Subjected to protease assay.

The extract of *C. luteum* at pH 9.0 was loaded on to a glass column coated with silica gel 60. The column was eluted with n-hexane, n-hexane–chloroform, and chloroform–ethyl acetate mixtures in different ratios to achieve 50 mL fractions consecutively. The fractions were subjected to thin-layer chromatography, and similar fractions were combined. The enzyme inhibition assay of each fraction was checked, and their results are presented in Table 2. The fractions collected in n-hexane-chloroform (10:90) were dried and treated to preparative TLC (ethyl acetate–methanol, 95:05), yielding colchicine (**1**) as a white solid (27 mg, 7.9 %age yield) as mentioned in Scheme 1 [25]. Rather et al., 2022 also reported colchicine in *Colchicum luteum* and quantified it using HPLC [26].

A sharp peak near 3200 cm^−1^ indicated the presence of N-H stretching vibration in the molecule. The ^1^H NMR spectrum of (**1**) exhibited an upfield singlet at δ 1.9, assigned to H-18, and four singlets at δ 3.6–3.9 due to the -OCH_3_ in the molecule. Three upfield multiplets at δ 2.2–4.49 were assigned to C-6, C-7, C-8, and two downfield doublets at 6.8–7.3 were due to C-10 and C-11 [27]. In ^13^C NMR spectrum, twenty two signals appeared, of which there were ten quaternary, five methine, two methane and five methyl carbons [25]. The ethanolic crude extract of *Camellia sinensis* dissolved in water and fractionated with n-hexane and chloroform, at acidic as well as at basic pH, successively. The extracts were further subjected to a protease inhibition assay according to the standard procedure (Table 3). The chloroform fraction at pH 9.0 showed an inhibitory activity 32.8 ± 0.4 age, which was further subjected to assay for isolation of bioactive compounds.

**Scheme 2:**
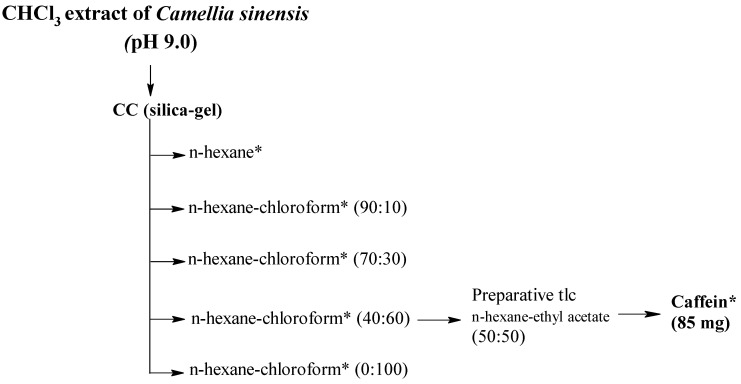
Isolation and purification of caffeine (**2**). * = subjected to protease assay.

Bioassay-guided column chromatography of the chloroform extract at pH 9.0 was carried out using a silica-packed column and it was eluted with solvent mixtures of n-hexane and chloroform. All column fractions were also assayed against protease inhibition activity (Table 4). The column chromatography fraction of chloroform extract (Scheme 2) at pH 9.0 obtained in n-hexane–chloroform (40:60) was dried and subjected to preparative TLC (n-hexane–ethyl acetate, 50:50), which yielded caffeine (**2**) as a white powder (85 mg, 12.6%age yield) [25].

The FTIR spectrum was characterized by a sharp peak near 3000 cm^−1^ and a strong signal at 1600 cm^−1^ due to N-H and C-O groups, respectively. ^1^H NMR spectra (CDCl_3_, 400 MHz) were characterized by three singlets at δ 3.23, 3.38, and 3.82, which corresponded to three methyl groups at C-12, C-11, and C-13, respectively. A downfield signal at δ 7.57 was assigned to the methine at C-2, which appeared at 142.5 in the ^13^C NMR spectrum [25].

**Scheme 3:**
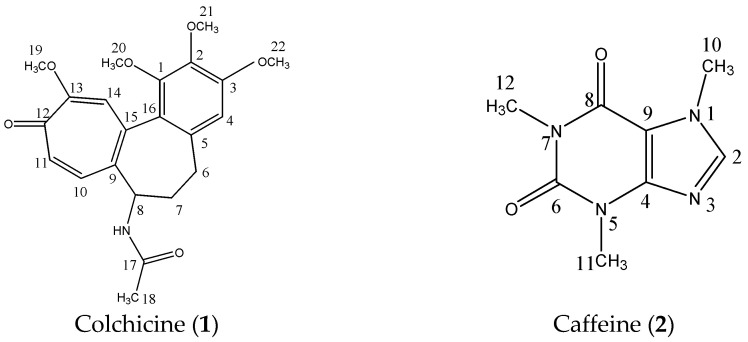
Purified compounds.

Colchicine (**1**) (Scheme 3)

IR (KBr, cm^−1^) υ_max_: 3280 (N-H), 2921 (C-H), 1623 (C=O), 1580 (C=C), 1070 (C-N); ^1^H NMR (400 MHz, DMSO): δ 1.93 (3H, s), 2.23 (2H, m), 2.47 (2H, m), 3.63 (3H, s), 3.93 (9H, s), 4.94 (1H, m), 6.54 (2H, s), 6.84 (1H, d), 7.24 (1H, d), 7.48 (1H, s). ^13^C NMR (100 MHz, DMSO d6): δ 21.4, 30.4, 38.7, 40.6, 41.2, 56.8, 60.9, 111.7, 119.8, 125.9, 128.2, 130.3, 132.0, 133.1, 139.1, 144.9, 153.0, 161.2, 172.9, 178.7, 185.0, 190.4.

Caffeine (**2**) (Scheme 3)

IR (KBr, cm^−1^) υ_max_ 3123 (N-H), 2970 (C-H), 1727 (C=O), 1552 (C=C), 979 (C-N); ^1^H NMR (400 MHz, CDCl_3_): δ 3.17 (3H, s), 3.35 (3H, s), 3.80 (3H, m), 7.60 (1H, s). ^13^C NMR (100 MHz, CDCl_3_): δ 21.4, 32.3, 37.3, 113.6, 127.9, 140.0, 148.9, 167.6. 

### 2.2. Protease Inhibitory Potential

In humans, several pathological conditions have been connected to disturbances in the function of proteases. So, they are considered a potent target for the design of new drugs or drug discovery [28]. It is reported that various protease inhibitors, such as bortezomib and dabigatran, are being made available in the market for the treatment of various diseases such as pulmonary embolism, atrial fibrillation, and hypertension. Nowadays, protease inhibitors are also being used clinically for the treatment of infectious diseases including HIV/AIDS and hepatitis C infections. In the present study, colchicine (**1**) was purified from a chloroform extract (pH 9.0) of the aerial parts of *C. luteum*, which showed remarkable protease inhibition activity with IC_50_ = 0.83 ± 0.07 mM (63.7 ± 0.5 %age inhibition at 100 µg/mL) compared with the standard (IC_50_ = 0.11 ± 0.02 mM, 87.2 ± 1.2 %age inhibition), as shown in Table 5 [25]. *C. luteum* is well known for its tropolone-type alkaloids, which contain an aromatic seven-membered cyclic hydroxyketone ring of a non-benzenoid type and exhibit a broad spectrum of physiological activities [29,30].

Colchicine has been shown to disrupt the mitotic spindle of dividing cells [31]. Colchicine has also been shown to have an anti-inflammatory effect, which is thought to be due to direct contact with microtubules [32]. It is also suggested that this action of colchicine is due to its irreversible inhibitory potential for tubulin polymerization, which prevents signal transduction, which is required for the activation of the NLRP3 inflammasome [32]. Colchicine effectively suppressed trypsin activity in our investigations, which can be attributed to its tropolone ring with a side chain, as shown in docking studies.

### 2.3. Estimation of the Kinetic Parameter

**Figure 1:**
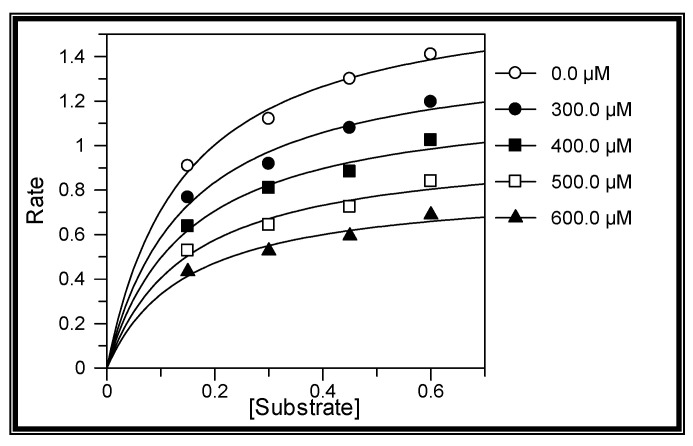
Rate of the reaction vs substrate at different concentrations of **1**.

**Figure 2:**
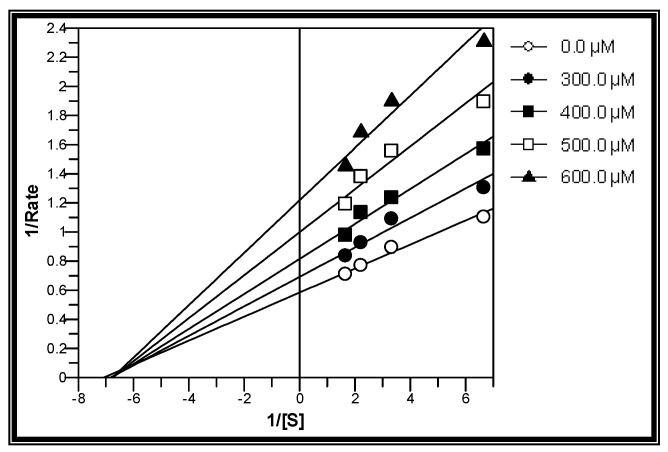
Lineweaver–Burk plot of reciprocal of rate vs reciprocal of fixed substrate concentration in absence and presence of different concentration of **1**.

**Figure 3:**
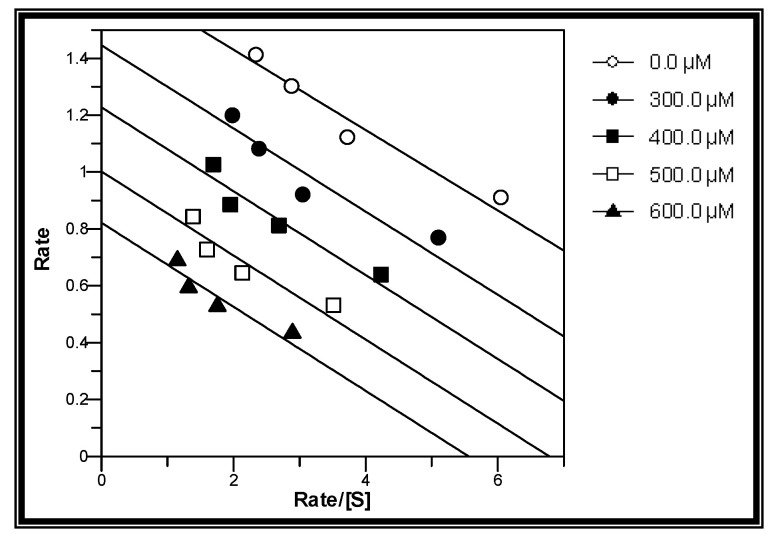
Rate of the reaction vs rate/substrate at different concentrations of **1**.

**Figure 4:**
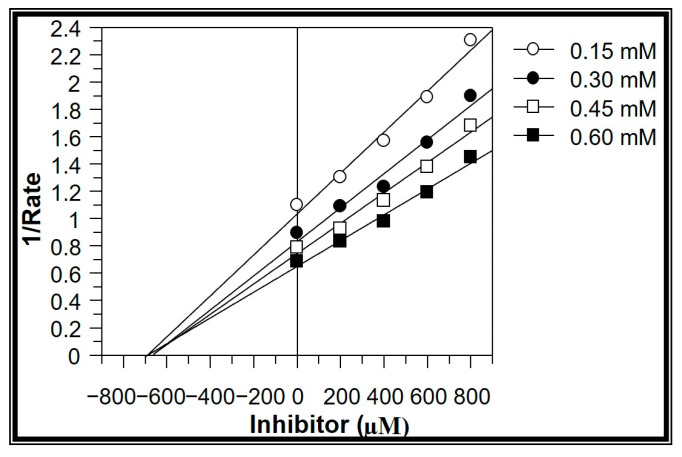
Dixon plot at fixed substrate concentrations.

**Figure 5:**
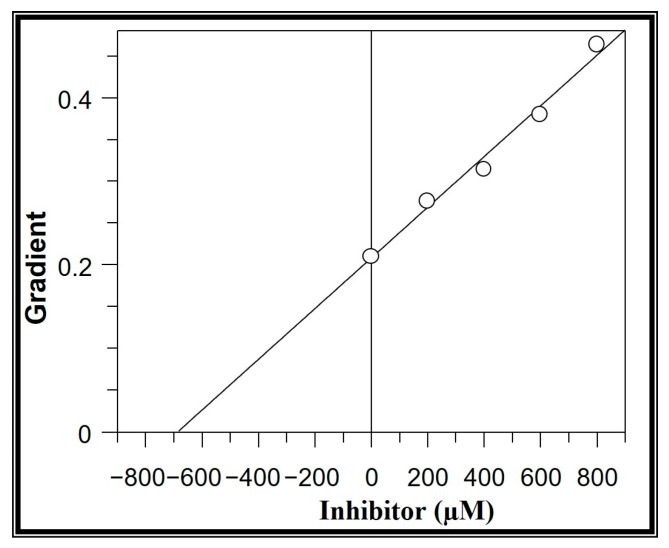
Lineweaver–Burk plot of gradient vs different concentrations of **1**.

**Figure 6:**
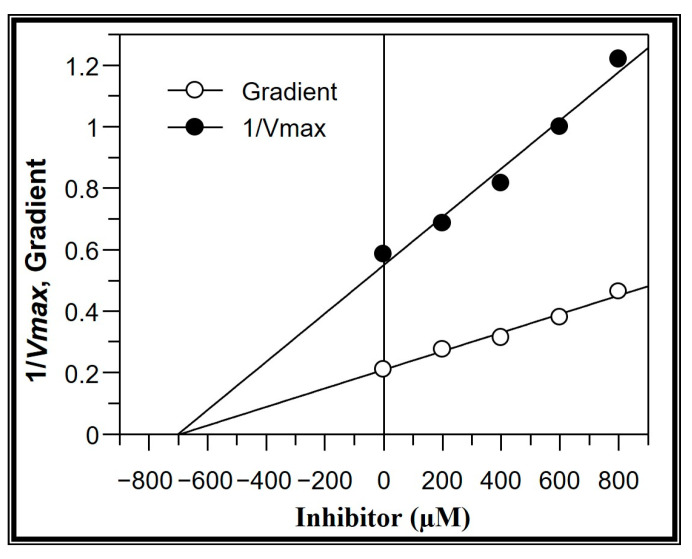
Secondary replots of the Lineweaver–Burk plot, *1/Vmax* and gradient vs various concentrations of **1**.

In order to determine the type of inhibition, two different methods were applied to monitor the effect of the inhibitor (test sample) on both *K_m_* and *V_max_* values (Figure 1, Figure 2, Figure 3, Figure 4, Figure 5 and Figure 6). First, the reciprocal of the rate of the reactions was plotted against the reciprocal of the substrate concentrations using the Lineweaver–Burk plot, and second, using the Dixon plot, in which the reciprocal of the rate of the reactions was plotted against the inhibitor concentrations [33,34]. In the case of the non-competitive and linear mixed-type inhibitions, the slope of each line of the inhibitor concentrations on the Lineweaver–Burk plot was plotted against the inhibitor concentrations. The secondary replot of the Dixon plot was constructed as the slope of each line of the substrate concentration in the original Dixon plot against the reciprocals of the substrate concentrations. The *K_i_* values (the dissociation constant of the dissociation of the enzyme–inhibitor complex into free enzyme and inhibitor) were determined by the interpretation of the Dixon plot, Lineweaver–Burk plot, and the secondary replots using initial velocities.

These velocities were obtained over a range of substrate concentrations between 0.14 and 0.56 mM. The assay conditions for the measurement of the residual activities of compound 1 were identical to the previously mentioned spectrophotometric assay procedure, except that fixed concentrations of the inhibiting compound were used in the assay medium. The type of inhibition was determined by the graphical views of the Dixon plots, Lineweaver–Burk plots, and their secondary replots [35,36,37]. It was noted from kinetic studies that colchicine (**1**) showed non-competitive behavior in trypsin inhibition, and a methyl group at position 3 was necessary for best activity.

### 2.4. In Silico Studies

**Figure 7:**
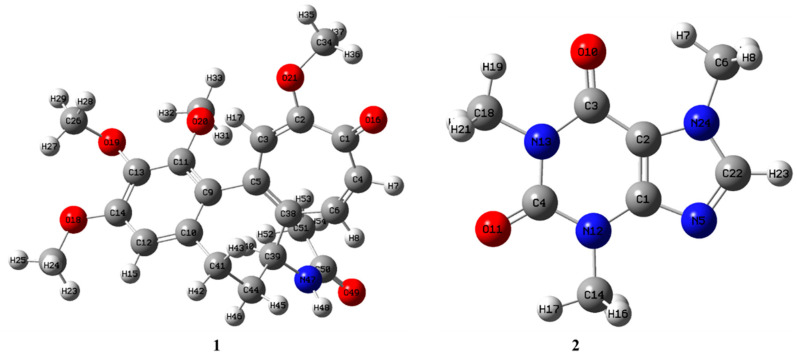
Optimized structures of the purified compounds.

**Figure 8:**
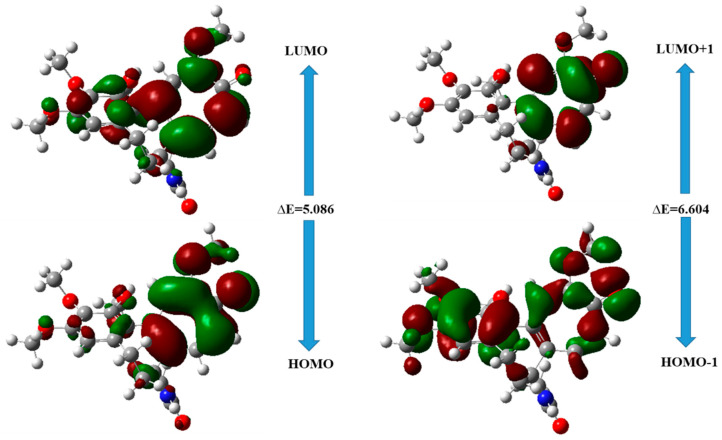
Frontier molecular orbitals of compound **1**.

**Figure 9:**
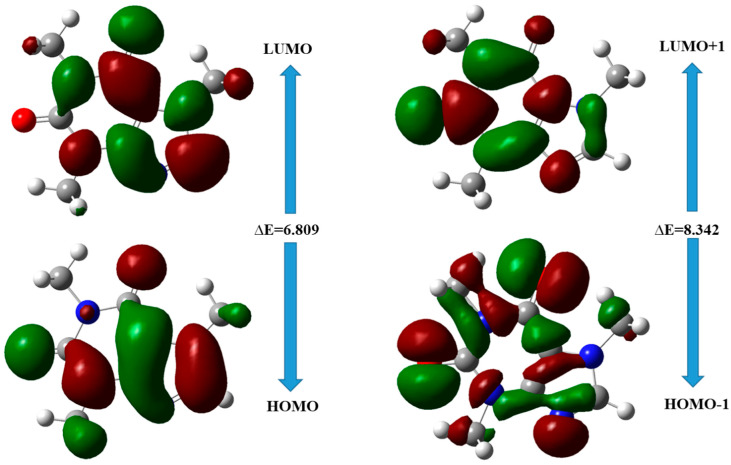
Frontier molecular orbitals of compound **2**.

We believe that theoretical studies currently receive great attention and can provide us with much information. Various software programs are available online that can predict the various properties of molecules. The purified compounds are optimized, as shown in Figure 7, with Gaussian software using a B3LYP functional and basis set of 6-31G(d,p). The output files are viewed in GaussView 6.0, and the HOMO/LUMO energy gap is calculated along with other parameters. The energy gap between HOMO and LUMO is 5.086 eV, while that between HOMO − 1 and LUMO + 1 is 6.604 eV for compound **1,** as shown in Figure 8. For compound **2**, the energy difference is 6.809 eV and 8.342 eV between HOMO/LUMO and HOMO − 1/LUMO + 1, respectively (Figure 9). The energy gaps of the two isolated compounds are enough to stabilize the molecules. Based on HOMO and LUMO, it was observed that electron density is located on the benzene rings of both compounds. The methyl groups present in both molecules have the least electron density, as depicted in the HOMO and LUMO figures.

**Figure 10:**
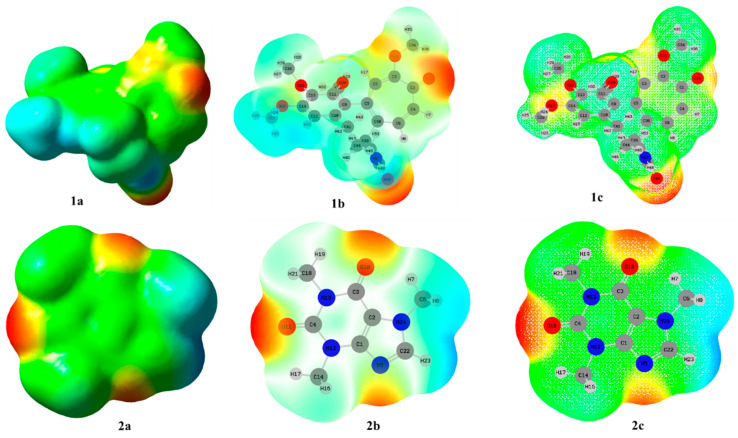
Molecular electrostatic potential of the purified compounds with different behaviors; (**a**) Total density, (**b**) Transparent, (**c**) Mesh format.

Density functional theory was also used to predict the molecular electrostatic potential (MEP) of the purified compounds, and helped with the reactivity of the molecule as a drug candidate. In MEP, plots were computed with different colors, which depicted the different behavior in terms of the potential of the molecule (Figure 10). The order of the different colors in terms of charge distribution on the surfaces was red > orange > yellow > green > blue [38]. The nitrogen (red) depicted negative potential, while the hydrogen (blue) of alkyl and aromatic rings showed positive potential. These may be utilized to detect physicochemical connections at the molecular level. Furthermore, the molecular electrostatic potentials along with other findings can be used to judge the chemical approachability of a new therapeutic agent to any electrophilic as well as nucleophilic attacks [39]. The red color shown on the surface of the molecule is due to the high electron density, and here any electrophilic moiety can be attached. These red areas may also be involved in the hydrogen bonding interaction during the attachment of the molecule as an enzyme inhibitor on the active site of the targeted enzyme. The blue areas on the surface show the electrophilic character of the molecule, where electron-rich species can interact.

Molecular docking is another tool to identify natural or synthetic molecules as therapeutic agents for curing different disorders [40]. Drug resistance is a hot issue now, and chemists and pharmacists are continuously working on the synthesis, purification, or design of new molecules to overcome this issue. Molecular docking studies are helpful in designing new molecules and to check their potency towards proteins [41]. To investigate the enzyme inhibition potential of the purified molecules on a theoretical basis, molecular docking studies were conducted against the PDB file of the protease. Interestingly, it was found that both compounds have the potential to bind to the active surfaces of the enzyme. Compound **1** showed good docking scores compared with compound **2** (Table 5). These results are also consistent with in vitro results showing that compound **1** is more effective than compound **2** against proteases.

**Figure 11:**
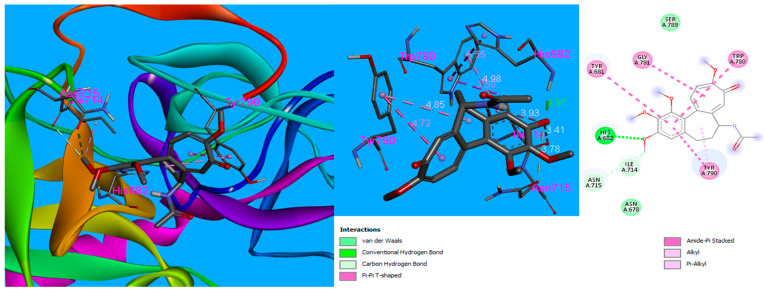
Molecular docking interaction of **1** on protease enzyme.

**Figure 12:**
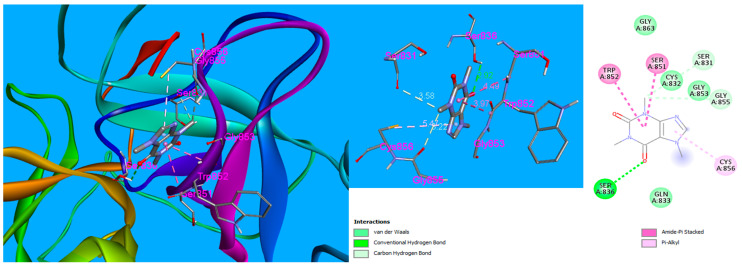
Molecular docking interaction of **2** on protease enzyme.

The colchicine compound (**1**) showed interactions with His682 through hydrogen bond interactions, with a distance of 1.96 Å, while weak interactions (pi-alkyl or pi-pi) were observed with Trp780, Gly781, Tyr681, and Tyr790, having distances of 3.59 Å, 5.55 Å, and 4.98 Å, respectively (Figure 11). Purified compound **2** also showed different interactions with different amino acids residues located on the surface of the targeted enzyme (Figure 12). The ketonic oxygen of compound **2** demonstrated hydrogen bond interactions with Ser836 (2.92 Å), while weak interaction in the form of Van der Wall were observed with Gly855, Cys856, and Ser831, with distances of 3.22,5.47, and 3.58, respectively, as shown in Figure 12. Similarly, π-alkyl interaction with Gly853 (3.97 Å) and with Ser851 (4.49 Å) were shown by compound **2**, as presented in Figure 12. These results suggest that purified compounds have the ability to bind with different amino acids on the active part of the protease and can inhibit the activity of the enzyme, which is necessary for the cure of various diseases caused by the overactivity of this enzyme.

## 3. Materials and Methods

### 3.1. Reagents and Instrumentation

Analytical-grade solvents such as methanol, ethanol, chloroform, ethyl acetate, n-hexane, and n-butanol were purchased from Panerac (Spain). Trypsin from bovine pancreas, N-benzoyl-DL-arginine-paranitroanilide-HCL (BApNA), and Silica Gel 60 were purchased from Fluka. Fourier-transform infrared spectra were run using KBr disks on a Perkin-Elmer 735B infrared spectrophotometer. ^1^H and ^13^C NMR spectra were recorded at 400 MHz and 100 MHz frequencies, respectively, using a Bruker Avance spectrometer. NMR samples were prepared in CDCl_3_ containing tetramethylsilane as an internal standard.

### 3.2. Collection and Bioassay Guided Fractionation of Colchicium Luteum

*Colchicium luteum* (the whole plant) was collected from hilly areas of Pakistan and identified at the Department of Botany, GC University, Lahore (GCU-BOT-083). The plant material (450 gm) was soaked in ethanol at room temperature for seven days and after that it was filtered and solvent was evaporated at reduced pressure using rotary evaporator yielding crude extract (2.1 gm). The crude extract was dissolved in water and subjected to partition chromatography with n-hexane and chloroform at pH 7.0, 3.0, and 9.0, according to Scheme S1 [5,25]. 

### 3.3. Collection and Bioassay Guided Fractionation of Camellia sinensis

The leaves of *Camellia sinensis* were purchased from a local market and identified at the Department of Botany, G.C. University, Lahore (voucher # GCU-BOT-726). The plant material was air dried, powdered, and extracted (250 g) with ethanol. The crude extract (1.21 g) was filtered, concentrated, and subjected to solvent extraction successively with n-hexane and CHCl_3_ at pH 3.0, 7.0, and 9.0 (Scheme S1). All the extracts were evaporated at reduced pressure using a rotary evaporator [25].

### 3.4. Protease Inhibition Assay

The protease inhibition potential of all the crude extracts, fractions, and purified compounds was assayed according to the spectrophotometric method [35,37,42]. A total of 0.3 mL of the enzyme (trypsin) and 100 L of the inhibitor (extract, fraction, or purified compound) were incubated at 37 °C for 15 min; later, 0.6 mM substrate (BApNA) was added, and the final volume was made up to 2.5 mL with Tris buffer. The reaction mixture was incubated at 37 °C for 30 min. The reaction was quenched by adding 100 L (30%) acetic acid and reading the absorbance at 410 nm using a UV/Vis spectrophotometer. Phenylmethanesulfonyl fluoride (PMSF) was used as a positive inhibitor, while buffer was used as a negative control. The experiment was performed three times, and the standard deviation (STD) was calculated using MS Excel software. The percentage inhibition was calculated using the following formula:

$$\%\;\mathrm{Inhibition}\;=\frac{\mathrm{Absorbance}\;(\mathrm{blank})\;-\;\mathrm{Absorbance}\;(\mathrm{test})}{\mathrm{Absorbance}\;(\mathrm{blank})}\times 100$$

### 3.5. In Silico Studies

In silico studies in term of density functional theory and molecular docking were carried out using respective software. A DFT study of the purified compounds was carried out with Gaussian 09 to calculate the quantum chemical predictions [43]. The outcomes files of the optimization were opened using GaussView 6.0. The energy was minimized by applying the hybrid functional method B3LYP and basis set 6-31G(d,p) [44]. The molecular docking study was executed using well-known software [43]. Serine protease with PBD code 1S0Q was downloaded and used for further studies, and a default force field was used for energy minimization. The docking outcomes files were examined with discovery Studio Visualizer and different interactions were visualized.

## 4. Conclusions

The present study was designed to evaluate the local flora for the treatment of different diseases related to proteases. The plants were selected based on their ethnobotanical uses and the presence of alkaloids in rich amounts after a proper literature survey. The plants after extraction were subjected to phytochemical screening, which suggested the presence of alkaloidal moieties, and fractionated into various fractions according to their polarity. The most active fraction of each plant was then subjected to column chromatography to isolate the major component in the targeted plants. The purified compounds were analyzed with spectral techniques. The compounds were screened against protease using an in vitro model, and it was found that colchicine (**1**) was more effective against protease compared with caffeine (**2**). The results were also compared with a standard inhibitor (PMSF). Both compounds were further optimized with Gaussian software, and, from the output files of the Gaussian, the frontier molecular orbital (FMO) and molecular electrostatic potential (MEP) were also mapped. These results suggested a sufficient energy gap between HOMO and LUMO for the stability of the molecules. The molecular docking studies were conducted to investigate the theoretical aspects of the compounds as enzyme inhibitors. The conducted study suggests that these compounds have abilities to interact with the targeted enzyme with different interactions. Based on our finding, it can be concluded that isolated molecules have the potential to inhibit protease and can be effective against different diseases related to this enzyme.

## Figures and Tables

**Table 1:** Protease inhibition activity of the extracts of *C. luteum*.

S. No	Extracts (250 µg/mL)	Protease Inhibition (%Age)
1	ethanol	49.7 ± 1.1
2	n-hexane	6.9 ± 0.3
3	chloroform at pH 7.0	24.6 ± 0.7
4	chloroform at pH 3.0	17.4 ± 0.3
5	chloroform at pH 9.0	54.1 ± 0.5

**Table 2:** Protease inhibition activity of the CC fractions of *C. luteum* (pH 9.0).

S. No	Fractions (100 µg/mL)	Protease Inhibition (%Age)
1	n-hexane	9.4 ± 0.2
2	n-hexane–chloroform (80:20)	11.9 ± 0.3
3	n-hexane–chloroform (30:70)	19.0 ± 0.3
4	n-hexane–chloroform (10:90)	57.4 ± 0.7
5	chloroform–ethyl acetate (85:15)	21.0 ± 0.5

**Table 3:** Protease inhibition activity of the extracts of *Camellia sinensis*.

S. No	Extracts (250 µg/mL)	Protease Inhibition (%Age)
1	ethyl alcohol	52.7 ± 1.0
2	n-hexane	0.0 ± 0.0
3	chloroform at pH 7.0	44.3 ± 0.6
4	chloroform at pH 3.0	26.5 ± 0.4
5	chloroform at pH 9.0	32.8 ± 0.4

**Table 4:** Protease inhibition activity of the CC fractions of *Camellia sinensis* (pH 9.0).

S. No	Fractions (100 µg/mL)	Protease Inhibition (%age)
1	n-hexane	0.0 ± 0.0
2	n-hexane–chloroform (90:10)	12.4 ± 0.3
3	n-hexane–chloroform (70:30)	8.6 ± 0.5
4	n-hexane–chloroform (40:60)	34.7 ± 0.2
5	chloroform	14.2 ± 0.4

**Table 5:** Protease inhibition activity and molecular docking studies of the purified compounds.

Sr. No	Compounds/ Standard	Protease Inhibition (%age)	IC_50_ (mM)	Docking Score (kcal/mol)
1	Colchicine (**1**)	63.7 ± 0.5	0.83 ± 0.07	-6.2
2	Caffeine (**2**)	39.2 ± 1.3	NA	-5.8
3	PMSF	87.2 ± 1.2	0.11 ± 0.02	NA

## Data Availability

Data is available on request.

## Supplementary Materials

The following supporting information can be downloaded at: https://www.mdpi.com/article/10.3390/molecules28062459/s1, Scheme S1: Extraction scheme for Colchicium luteum and Camellia sinensis.
