# Supplementary material for: Bioassay-Guided Alkaloids Isolation from Camellia sinensis and Colchicum luteum: In Silico and In Vitro Evaluations for Protease Inhibition
Source: Molecules. 2023 Mar 8;28(6):2459. doi: 10.3390/molecules28062459 (PMC10058905; doi:10.3390/molecules28062459)
Supplement: Supplementary file 1 [file molecules-28-02459-s001.zip › molecules-2184427-supplementary.pdf]

Bioassay-Guided Alkaloids Isolation from *Camellia sinensis* and *Colchicum luteum*: *In Silico* and *In Vitro* Evaluations for Protease Inhibition

Mohammad Aatif [1,*], Muhammad Asam Raza [2,*], Mohamed El Oirdi [3], Mohd Farhan [3], Muhammad Waseem Mumtaz [2], Muhammad Hamayun [2], Adnan Ashraf [4] and Ghazala Muteeb [5]

1 Department of Public Health, College of Applied Medical Sciences, King Faisal University, Al Ahsa 31982, Saudi Arabia
2 Department of Chemistry, Hafiz Hayat Campus, University of Gujrat, Gujrat-50700, Pakistan
3 Department of Basic Sciences, Preparatory Year Deanship, King Faisal University, Al Ahsa 31982, Saudi Arabia
4 Department of Chemistry, University of Lahore, Lahore 53700, Pakistan
5 Department of Nursing, College of Applied Medical Sciences, King Faisal University, Al Ahsa 31982, Saudi Arabia
* Correspondence: maahmad@kfu.edu.sa (M.A.); asam.raza@uog.edu.pk (M.A.R.)

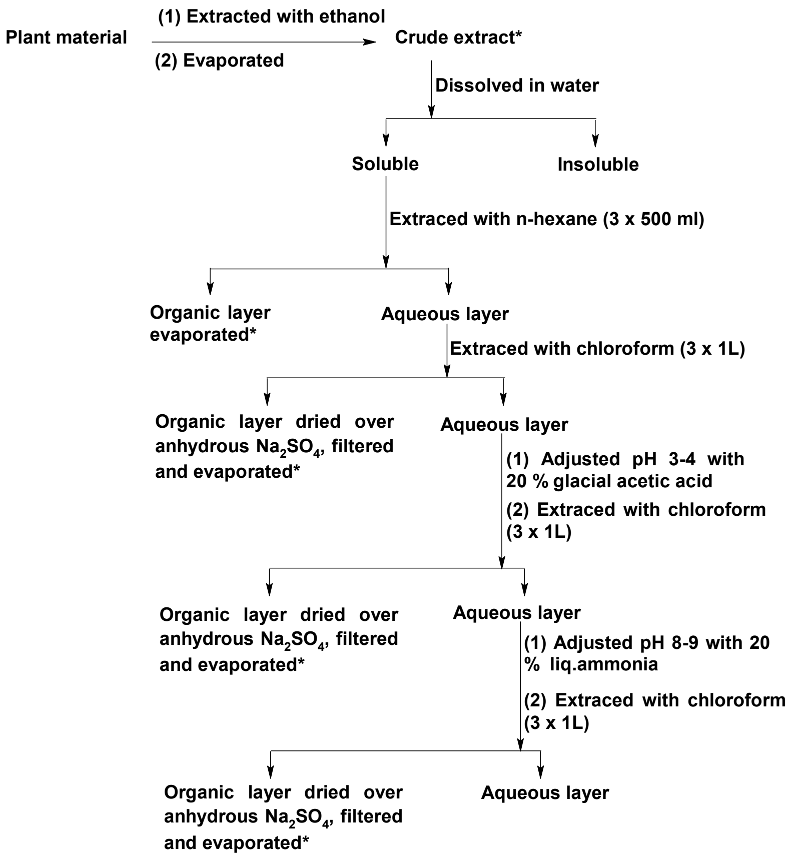

* = Subjected to protease assay

Scheme S1: Extraction scheme for *Colchicium luteum* and *Camellia sinensis*
